# Target Identification with Improved 2D-VMD for Carrier-Free UWB Radar

**DOI:** 10.3390/s21072465

**Published:** 2021-04-02

**Authors:** Yuying Zhu, Shuning Zhang, Huichang Zhao, Si Chen

**Affiliations:** School of Electronic and Optical Engineering, Nanjing University of Science and Technology, Nanjing 210094, China; zhuyuying@njust.edu.cn (Y.Z.); zhaohch@mail.njust.edu.cn (H.Z.); chensi354@njust.edu.cn (S.C.)

**Keywords:** carrier-free ultra-wideband radar, 2D-IVMD, transfer learning, DCNN multi-views signals

## Abstract

In recent years, the interest in radar automatic target recognition (RATR) based on the carrier-free ultra-wideband (UWB) radar has been increasing. Compared with narrow-band and other bandwidth radars, the echo signal of the carrier-free UWB radar includes more comprehensive and detailed information with respect to the targeted object. In this paper, we first utilized 3ds Max to acquire accurate geometric models and applied a time-domain integral equation (TDIE) for echo signal acquisition under the condition that the transmitted signals had an extremely short duration period. By comparing the simulated waveform with the actual one, the accuracy of the electromagnetic modeling is verified. Furthermore, given that the actual environment is full of noise and clutter, we propose an improved two-dimensional variational mode decomposition (2D-IVMD), and an algorithm is proposed to eliminate noise and extract edge features preliminarily, which lays a foundation for further in-depth feature extraction. Then, the deep conventional neural network (DCNN) is introduced for the final recognition. The results show that the proposed methods achieve promising classification performance under the condition of low signal-to-noise ratio (SNR) values.

## 1. Introduction

Carrier-free ultra-wideband (UWB) radar, which transmits signals with a pulse width of nanoseconds and a large bandwidth from direct current to gigabit, possesses high spatial and temporal resolution [1]. The aforementioned properties suggest that the UWB radar has some optimality in operating target identification and detection, e.g., personnel positioning [2], ground penetrating [3], and human motion recognition [4]. Even though the UWB echo signal can provide plenty of information about targets, using the carrier-free UWB radar to recognize large complex targets is still a challenging task since there are only a few research documents with respect to it. One of the main reasons is that the lack of research on the electromagnetic scattering characteristics of complex targets makes it hard to collect sufficient reflected waveforms from targets; moreover, the extreme within-class difference with the target-sensor orientation variation is also a notable nodus.

One of the critical issues of the target classification is to accurately model the objective and obtain a large amount of echo data. Generally speaking, there are three major strategies to achieve this goal: practical measurement, scaled experiment, and electromagnetic (EM) simulation; however, the first two methods are not only expensive but also struggle to acquire measured non-cooperative signals. Some researchers have conducted extensive studies on various EM calculation methods for target scattering characteristics. Analytical solutions can provide precise results but only for simple regular objects [5]. High-frequency methods fail to take all EM phenomena into account [6,7,8]. Considering all EM effects, the full-wave technique is widely used in EM scattering studies for complex targets [9]. Moreover, time domain methods can obtain broadband data through only one calculation and clearly reflect the entire physical process, which differs from frequency domain methods. The finite-differential time-domain (FDTD) method [10] and the finite-element time-domain (FET) method [11] have advantages in processing inhomogeneous materials; however, dividing the whole propagation space will generate a large computational domain. On the contrary, the time-domain integral equation (TDIE) [12,13] is more suitable for solving open region problems since the solution of the integral equation automatically satisfies the radiation boundary condition.

In many applications of machine learning, feature extraction is fundamental for different systems. Classification performance based on UWB signals suffers from the target aspect, time shift, and amplitude sensitivity. To overcome these problems, some researchers pay a lot of attention to exploring time-shift invariance features and conduct robust feature templates. Making use of the invariance property of the Mellin transform, features that are insensitive to the aspect angle of targets were extracted [14]. Time-shift invariance features, such as bispectra [15] and higher-order spectra [16], were applied to target classification. Since the high dimensionality of UWB echo data causes a big burden to the storage system and the classifier, some dimensional reduction techniques were proposed, such as linear discriminant analysis (LDA) [17] and principal component analysis (PCA) [18]. However, such methods based on one-dimensional signals can only make use of limited information, which decreases the identification performance. Additionally, most of them mainly aim at UWB signals with carrier frequency instead of carrier-free waveforms.

In order to acquire more information from the data, there are more applications that we are interested in performing target identification based on a sequence of echo signals. One approach to processing these waveforms is to form images in which the variance of strong scattering points is extracted as input features. Before image-based target classification, image preprocessing is necessary, such as image denoising and preliminary edge feature extraction. Recently, various spectral decomposition methods have been extensively investigated, which include short-time Fourier transform (STFT) [19], continuous waveform transform (CWT) [20], and basis pursuit (BP) [21]. Though these methods have had success regarding simple signals, they are still not fully robust to non-stationary waves. Empirical mode decomposition (EMD) [22,23,24] proposed by Huang et al. is a self-adaptive signal processing method, which is preferable for the non-linear and non-stationary signal; however, shortcomings, such as the lack of mathematical theory, mode-mixing phenomenon, and sensitivity to noise, have restrained the application and its development. To settle these limitations of the original EMD, Dragomiretskiy and Zosso proposed a variational mode decomposition (VMD) [25], which is fully intrinsic and adaptive. In the application of decomposition for 2D signals, 2D-VMD [26], decomposing a 2D signal into several modes with different center frequencies, and holding a Wiener filter property, makes it possible to denoise and extract edge features at the same time.

In recent times, deep learning algorithms have gained a lot of popularity in image classification and identification. Typical deep network architectures include the restricted Boltzmann machine (RBM) [27], stacked denoising autoencoder (SDAE) [28,29], and convolutional neural network (CNN) [30,31]. Some studies have verified the effectiveness of SDAE and RMB, but unlike CNN, they cannot be invariant to transformations. Therefore, SDAE and RBM fail to deal with the problems of target aspect and translation sensitivity that arise when using carrier-free UWB signals for target classification. Traditional DCNNs must be fed a vast amount of data to obtain the optimal model. However, aiming at non-cooperative targets, incomplete data will lead to the failure of majorizing model parameters and ultimately affect the classification results. In response to this problem, many researchers utilized transfer learning to enhance models’ learning efficiency. Transfer learning shares trained parameters to new models with full consideration of the tight relation among most of the tasks and helps to save calculation time. Table 1 shows the pros/cons of the state-of-the-art methods.

In this study, we first apply the carrier-free UWB signals for complex target recognition. Due to the extreme bandwidth of free-carrier UWB signals, 3ds Max and TDIE are introduced for accurate geometric modeling and echo signal acquisition, respectively. An improved 2D-VMD method is proposed. The key novelty of this method is that, by means of selecting appropriate 2D-intrinsic modes, it is not only used in feature extraction, but also for 2D signal/image denoising and sharpening, which lays a foundation for further feature extraction by using CDNNs. The experimental results show that the proposed method is effective for target classification in the case of a low signal-to-noise ratio (SNR) from −10 to 2 dB.

The paper is organized as follows: Section 1 makes a brief introduction. The EM models for targets are elaborated in Section 2. Section 3 describes the theoretical basis and application in the ground targets identification of the two-dimensional variational mode decomposition (2D-IVMD). The transfer learning based on the deep conventional neural networks (CDNNs) is introduced in Section 4. Experimental results illustrating the classification performance of the proposed methods are presented in Section 5. Finally, Section 6 presents the summary and the outlook of the article.

## 2. Database Construction

Generally, a target classification database is composed of measured data [32,33]. Researchers validate their methods by comparing actual echoes with previously collected data of a potential target. However, since it is impossible to obtain the fill echo data of enemy targets, populating a database through EM simulation is necessary [34,35]. EM calculation methods have both strengths and weaknesses. The advantages are their cost and time efficiency, and their ability to obtain the signature of any angles in their entirety. However, they only work under ideal simulation environments, not all situations are considered, and CAD models have insufficient accuracy. In response to these issues, 3d Max and TDIE were, respectively, introduced for accurate target modeling and target echo acquisition. The measured simulated signals based on a simple object were compared to demonstrate the effectiveness of the modeling method.

### 2.1. The TDIE Algorithm

The finite integration technique in time domain is a valuable tool for solving complex industrial electromagnetic problems. This numerical method provides a universal spatial discretization scheme applicable to various electromagnetic problems ranging from static field calculations to high-frequency applications in the time or frequency domain. Unlike other numerical methods, TDIE is based on the integral form of Maxwell’s equation rather than the differential one:

(1) $$\oint_{C}\vec{H}(\vec{r},t)\cdot d\vec{l}=\int_{S}\left(\vec{J}(\vec{r},t)+\frac{\partial \vec{D}(\vec{r},t)}{\partial t}\right)\cdot d\vec{S}$$

(2) $$\oint_{C}\vec{E}(\vec{r},t)\cdot d\vec{l}=\int_{S}\left(\frac{\partial \vec{B}(\vec{r},t)}{\partial t}\right)\cdot d\vec{S}$$

(3) $$\oint_{S}\vec{B}(\vec{r},t)\cdot d\vec{S}=0$$

(4) $$\oint_{S}\vec{D}(\vec{r},t)\cdot d\vec{S}=\int_{V}\rho (\vec{r})d\vec{r}$$

where S is the surface of the object and V is the dielectric field. $\vec{E}(\vec{r},t)$ represents the transient electric field, $\vec{D}(\vec{r},t)$ the transient displacement field, $\vec{B}(\vec{r},t)$ the transient magnetic field, and $\vec{H}(\vec{r},t)$ the transient magnetizing field. $J(\vec{r},t)$ and $\vec{E}(\vec{r},t)$, respectively, refer to an electric current density and a charge density distribution.

Discrete forms of the Maxwell equation are the foundation of the TDIE method covering all kinds of EM effects; thus, the following section shows the derivation of discrete forms of the Maxwell equation.

**Figure 1:**
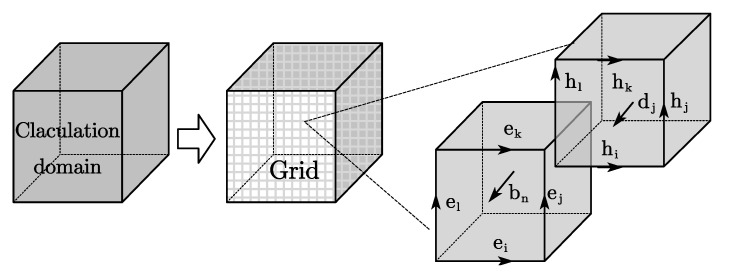
Maxwell’s grid division.

As shown in Figure 1, the calculation domain was firstly divided into N subdomains, and then, the initial discretization of Maxwell’s equation was performed on the two orthogonal grid systems. The electric grid voltages and magnetic facet fluxes were allocated on the primary grid, and the dielectric facet fluxes as well as the magnetic grid voltages were defined on the dual grid. Vectors can be respectively defined as

(5) $$e=\oint_{C}\vec{E}(\vec{r},t)\cdot d\vec{l}$$

(6) $$b=\int_{S}\vec{B}(\vec{r},t)\cdot d\vec{S}$$

By introducing vectors e and b, Equations (2) and (3) can be rewritten as matrix equations:

(7) $$Ce=-\frac{d}{dt}b$$

(8) Sb=0

In the same way, Equations (1) and (4) can be rewritten as:

(9) $$\tilde{C}h=J+\frac{d}{dt}d$$

(10) $$\tilde{S}b=q$$

where the expressions of h, d, J, and q are as follows:

(11) $$h=\oint_{C}\vec{H}(\vec{r},t)\cdot d\vec{l}$$

(12) $$d=\int_{S}\frac{\partial \vec{D}(\vec{r},t)}{\partial t}\cdot d\vec{S}$$

(13) $$J=\int_{S}\vec{J}(\vec{r},t)\cdot d\vec{S}$$

(14) $$q=\int_{V}\rho (\vec{r})d\vec{r}$$

where both matrices C and $\tilde{C}$ represent the discrete curl operator ∇×, and S and $\tilde{S}$ are the discrete divergency operator ∇·.

The set of Equations (7)–(10) is Maxwell’s grid equation. By means of solving this equation, we can drive most EM problems, e.g., for electrostatics, magnetostatics, transient scattering problems, and high-frequency issues.

### 2.2. Comparative Experiments

To justify the accuracy and robustness of the modeling method, experiments making comparisons between the measured echo and simulated signal were conducted, and two targets with diverse properties (i.e., material, size and geometry) were analyzed. They were a conductor sphere and triangular reflector. Although it is a simple shape, a sphere is still a difficult target for target recognition because of its complex resonating responses.

**Figure 2:**
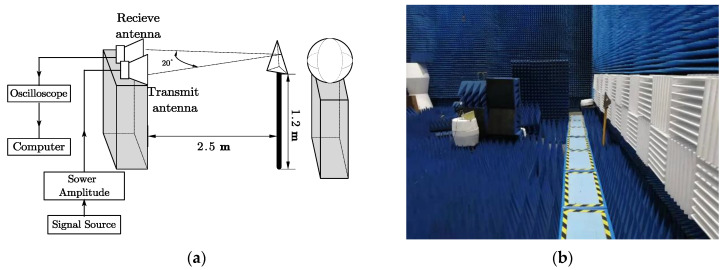
Experimental facilities layout: (**a**) schematic diagram and (**b**) practical measurement diagram.

To demonstrate the proposed method’s effectiveness, a series of experiments were designed for the two targets: a conductor sphere with a diameter of 0.6 m and a triangular reflector with a side length of 0.3 m. We studied the echo signals of the two targets separately. Figure 2 depicts the layout of the experimental facilities in a microwave anechoic chamber. Both the transmit antenna and the receive antenna are double-ridged horn antennas with an operating frequency of 0.5–18 GHz, which have excellent directivity. They were placed on a high platform 0.9 m above the ground, and 1.3 m apart horizontally. The middle was filled with absorbing materials to eliminate ground clutter and coupling waves between the antennas. The working procedure was as follows: first, the signal source transmitted a signal with a 2.5 nanosecond duration time and 0.5–3 GHz bandwidth, which was then amplified by the power amplifier, set out by the transmit antenna, and accepted by the receiving antenna. Finally, the target scattering echo was displayed in the oscilloscope and saved on the computer.

**Figure 3:**
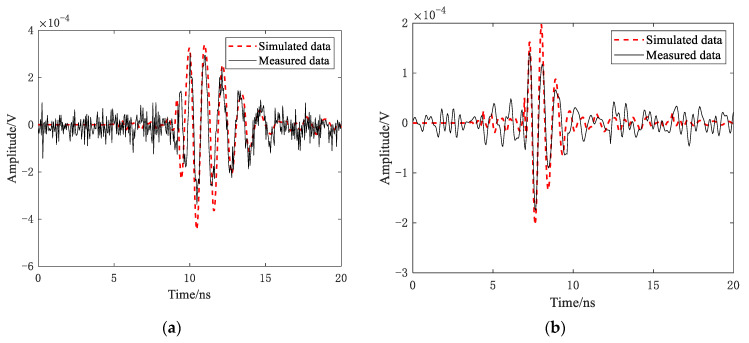
Experiment results: (**a**) triangular reflector and (**b**) conductor sphere.

In analogy to the measurement experiment settings (e.g., locations, target parameters, and antenna structures), simulations were also run for these targets. Comparative results are shown in Figure 3. It was clear that the measured echo carried noise, while the simulation did not; however, major parts of the target echo were almost the same, which demonstrates the accuracy of the EM modeling methods.

### 2.3. Ground Targets Modeling

**Figure 4:**
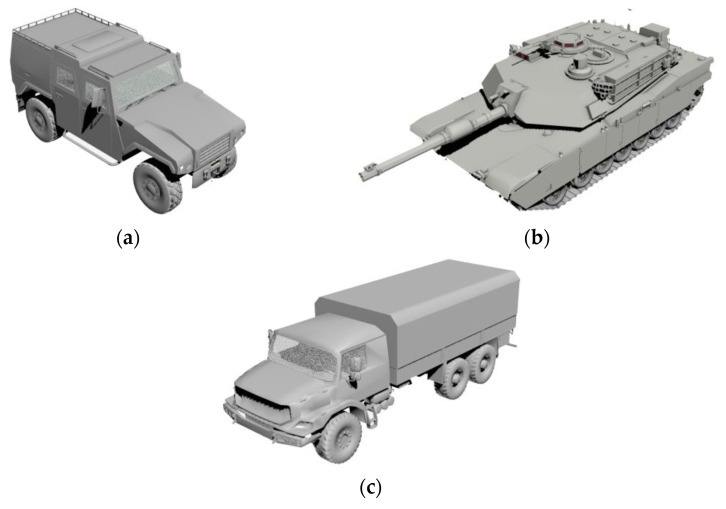
Geometric models: (**a**) wheeled vehicle; (**b**) tracked car; (**c**) box truck.

The prerequisite for obtaining a precise target echo is an accurate geometric model. To achieve this purpose, 3ds Max was employed to construct geometric models of three vehicles. The three typical targets are wheeled vehicles, tracked cars, and box trucks. The optical images are shown in Figure 4, which restores the details and contours of the vehicles as much as possible. After obtaining the geometric models, the waveform libraries for the three typical ground targets were established by implementing the TDIE method. For simplicity, we assume that the target-sensor orientations were modeled as a constant in the depression angle, while the azimuthal angles were not fixed. To testify the robustness of the classification method, the training set data were observed at a depression angle of 30°, and the test set was observed at 25°. Moreover, azimuth angles varied continuously from 0 to 180° with an interval of 0.3°.

**Figure 5:**
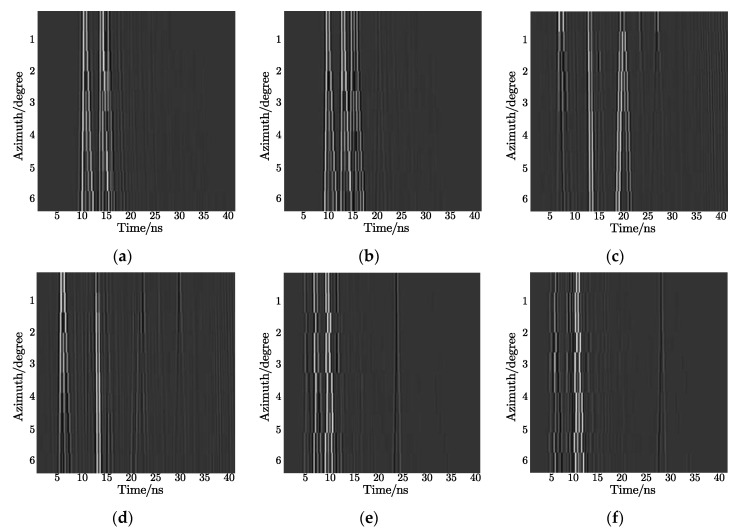
Geometric models: (**a**) wheeled vehicle with 25° azimuth; (**b**) wheeled vehicle with 30° azimuth; (**c**) tracked car with 25° azimuth; (**d**) tracked car with 30° azimuth; (**e**) box truck with 25° azimuth; (**f**) box truck with 30° azimuth.

Considering that multi-views contain more target signatures, we used the 2D signal consisting of a series of echo pulses with continuously varying azimuthal angles for feature extraction. However, obtaining target echoes in an extensive-angle range is unrealistic; therefore, signals within a certain angle were selected to form a 2D echo. As shown in Figure 5, each 2D signal has 20 echo pulses corresponding to the 20 azimuth angles from 0 to 6° for each target, and there was an apparent difference between the 25° azimuth and the 30° azimuth. Additionally, the reason why 2D signals (which differ from simple 1D echoes) can improve the recognition rate is that the varying trend of the strong scattering points with azimuth angles, instead of single position information, is used as input features.

## 3. Improved 2D-VMD Algorithm

### 3.1. 2D-VMD Algorithm

The 2D-VMD is a new adaptive time-frequency analysis tool. It decomposes a 2D analytical signal into an ensemble of modes to realize the effective separation of signals from low- to high-frequency, where each mode has a limited bandwidth and characteristic center frequency. With the intent of obtaining corresponding modes [28], the variational constraint model of the 2-dimensional signal f(x) was constructed by the following steps:

Step 1: Calculate the analytical signal by 2D Hilbert transform. According to the characteristics of Fourier transform, the 2D analytical signal $u_{AS,k}(x)$ is defined as:

(15) $$u_{AS,k}(x)=u_{k}(x)*\left(\delta \left(\langle x,\omega_{k}\rangle \right)+\frac{j}{\pi \langle x,\omega_{k}\rangle }\right)\delta \left(\langle x,\omega_{k,⊥}\rangle \right)$$

where $u_{k}(x)$ is the 2D intrinsic mode and $\omega_{k}$ is the center frequency corresponding to each mode. δ(x) denotes the unit impulse function and ∗ represents the convolution operation.

Step 2: Shift the frequency spectrum to the corresponding frequency baseband by complex harmonic mixing.

(16) $$u_{AS,k}(x)\times e^{-j(x,\omega_{k})}$$

Step 3: Estimate the bandwidth of each mode by calculating the square of the demodulated signal gradient $L_{2}$ norm.

(17) $${\left\|\nabla u_{AS,k}(x)\times e^{-j(x,\omega_{k})}\right\|}_{2}{}^{2}$$

Step 4: Search K modes to minimize the sum of the bandwidths of all intrinsic modes, and the variational constraint form of the 2D analytical signal is eventually represented as:

(18) $$\begin{array}{l} \underset{u_{k,}\vec{\omega_{k}}}{\min}\left\{\sum_{k}{\left\|\nabla u_{AS,k}(x)\times e^{-j(x,\omega_{k})}\right\|}_{2}{}^{2}\right\}\\ s.t.\;\sum_{k}u_{k}=f \end{array}$$

To solve the objective function in Equation (18), the penalty parameter α and the Lagrangian multiplier λ(x) are introduced. These two parameters render the problem unconstrained and guarantee the accuracy of the signal reconstruction in the presence of noise. The augmented Lagrangian function is as follows:

(19) $$\begin{array}{ll} L\left(\left\{u_{k}\right\},\left\{\omega_{k}\right\},\lambda \right) & =\alpha \sum_{k=1}^{K}{\left\|\nabla u_{AS,k}(x)\times e^{-j(x,\omega_{k})}\right\|}_{2}{}^{2}\\ & +{\left\|f-\sum_{k=1}^{K}u_{k}(x)\right\|}_{2}{}^{2}\\ & +\langle \lambda (x),f-\sum_{k=1}^{K}u_{k}(x)\rangle \end{array}$$

The alternate direction method (ADMM) is introduced to deal with the unconstrained problem. By updating iteratively $u_{k}{}^{n+1}$, $\omega_{k}{}^{n+1}$ and $\lambda_{k}{}^{n+1}$, the saddle points of the Equation (19) are found, and then the optimal solutions can be obtained. The expression of $u_{k}{}^{n+1}$ is depicted as:

(20) $$u_{k}{}^{n+1}(\omega )=\frac{f(\omega )-\sum_{i\neq k}u_{i}(\omega )+\frac{\lambda (\omega )}{2}}{1+2\alpha {\left(\omega -\omega_{k}\right)}^{2}}$$

Similarly, the optimal solution for the center frequency $\omega_{k}^{n+1}$ is expressed as:

(21) $$\omega_{k}{}^{n+1}=\frac{\int_{\Omega_{k}}\omega {\left|u_{k}(\omega )\right|}^{2}d\omega }{\int_{\Omega_{k}}{\left|u_{k}(\omega )\right|}^{2}d\omega }$$

where $\omega \in \Omega_{k},\Omega_{k}=\left\{\omega |\omega \cdot \omega_{k}\geq 0\right\}$.

In summary, the specific process of the 2D-VMD algorithm is illustrated as follows:

(a) Initialize $\left\{u_{k}{}^{1}\right\}$, $\left\{\omega_{k}{}^{1}\right\}$ and n.
(b) Update $u_{k}$ and $\omega_{k}$ in the frequency domain according to Equations (20) and (21).
(c) Update λ(x), where
  (22) $$\lambda^{n+1}(\omega )=\lambda (\omega )+\tau \left(f(\omega )-\sum_{k=1}^{K}u_{k}{}^{n+1}(\omega )\right)$$
(d) Stop iteration when $\sum_{k=1}^{K}\frac{{\left\|u_{k}{}^{n+1}-u_{k}{}^{n}\right\|}_{2}^{2}}{{\left\|u_{k}{}^{n}\right\|}_{2}^{2}}<e$, where e satisfies e>0; otherwise, return to step (b).

### 3.2. The Improved 2D-VMD Algorithm

Due to the tight relations to the Wiener filter, the 2D-VMD is often used for image denoising [36,37], which lays a foundation for extracting in-depth features using CNNs. However, the denoising performance strongly depends on the value of K and the modes combination. Moreover, the procedure of filtering the high-frequency noise will blur the edges of the image, which will be harmful to further feature extraction.

The 2V-VMD algorithm divides the input image into several modes and a residue. The expression can be formulated as follows:

(23) $$X(x,y)=\sum_{i=1}^{K}IMF_{i}(x,y)+r(x,y)$$

where X(x,y) is the input image with noise and r(x,y) represents the residue.

The K intrinsic modes contained image information from low- to high- frequency, and the residue mostly included high-frequency noise. In fact, effective decomposition of 2D signals from low-frequency to high-frequency suggests that the 2D-VMD algorithm has optimality in dealing with image sharpening. In other words, discarding the residual and low-frequency components can achieve the purpose of image denoising and contour extraction at the same time.

Unlike the traditional 2D-VMD method, in the improved 2D-VMD algorithm, we discarded the residue to filter noise and certain low-frequency IMFs to highlight the edges of input images. Since the critical point of this process lies in the selection of K, it raised the question on how to define the number of modes; however, few studies have been conducted on the selection of this parameter for the 2D-VMD algorithm.

There are two aspects that must be considered during modes selection. On the one hand, with the intent to sharpen the image by filtering low-frequency components, K must be greater than 1; on the other hand, a value that is too large will increase noise and time consumption. In this paper, a new method is proposed to define K by analyzing the 2D spectrum of the original signal and the noisy signal.

**Figure 6:**
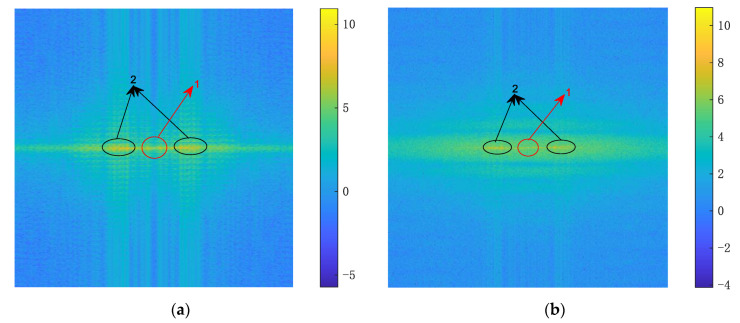
2D frequency spectrum: (**a**) 2D frequency spectrum of the original signal and (**b**) 2D frequency spectrum of the noisy signal, where signal-to-noise ratio (SNR) is −2 dB.

The amplitude spectrum of the useful signal and the noisy signal, where we chose an SNR = −2 dB, is shown in Figure 6. The frequency spectrum of the useful signal mainly consists of two parts: the middle, which includes the lowest frequency composition and can be considered as the textures of a 2D-signal/image, and the medium frequency composition, which tends to occur at image edges. Furthermore, when compared with Figure 6a, the redundant parts in Figure 6b represent noise. According to the above analysis, for ground target identification, the 2D echo is decomposed into two modes: BIMF1 and BIMF2. BIMF1 mainly represents the texture information of a 2D signal, while BIFM2 comprises the edge information. By only preserving BIMF2 can the image edge be extracted, which highlights the fluctuating trend of strong scattering points with azimuths.

Comparing the peak signal noise rate (PSNR) between the original 2D echo signal without noise and the reconstructed 2D echo signal with a different SNR further illustrates the rationality of the decomposition layers as two. As can be seen in Table 2, the larger the value of K, the smaller the PSNR and the worse the noise filtering performance. The PSNR is expressed as follows:

(24) $$\begin{array}{l} MSE=\frac{1}{H\times W}{\sum_{i=1}^{H}\sum_{j=1}^{W}\left(X\left(i,j\right)-Y\left(i,j\right)\right)}^{2}\\ PSNR=10\log_{10}(\frac{{\left(\max\right)}^{2}}{MSE}) \end{array}$$

where MSE is the mean square error of the original 2D signal X(i,j) and the reconstructed 2D signal Y(i,j); H and W are the height and the width of the signal, respectively; max is the maximum value in the 2D matrix.

**Figure 7:**
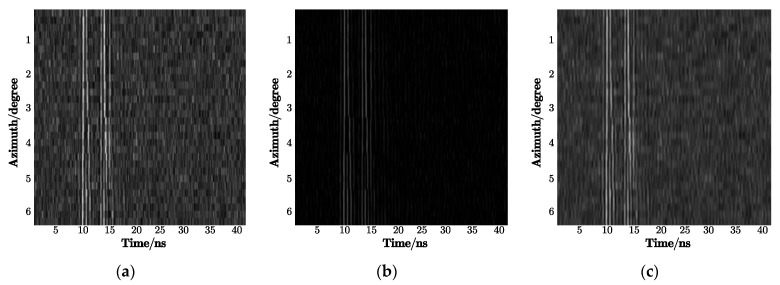
2D echo signal: (**a**) the original echo with noise, where SNR equals −2 dB; (**b**) decomposed mode BIMF2; (**c**) the sum of BIMF1 and BIMF2.

In the 2D-IVMD algorithm, using BIMF2 as the input image for target recognition solved the problem of image blurring, and it laid the foundation for using DCNN to extract in-depth features. Figure 7a shows the 2D echo in the case of SNR = −2 dB, Figure 7b shows the decomposed mode BIMF2, and Figure 7c shows the sum of BIMF1 and BIMF2. It can be seen that the sharpened image not only sufficiently filters out the noise but also highlights the direction of strong scattering points. In contrast, the image including BIMF1 saves texture information while weakening profiles, which makes the figure visibly unclear.

## 4. Deep Convolutional Neural Network

The key to deep learning is sufficient data. For example, the AlexNet proposed in 2012 was trained by the database consisting of more than one thousand objects and one million images. Otherwise, such a vast amount of data is almost impossible to access in the practical application. How can we make full use of these trained models instead of starting from scratch? As its name implies, transfer learning refers to the use of existing knowledge to learn new knowledge. Different from traditional machine learning, transfer learning exploits the similarities between tasks to perform knowledge transformation. The most common operation is to use the weights of the trained DCNN as initial settings or a fixed feature extractor for the related task. In this section, the convolution neural network VGG16 (Visual Geometry Group) proposed in 2014 is introduced for ground target classification.

**Figure 8:**
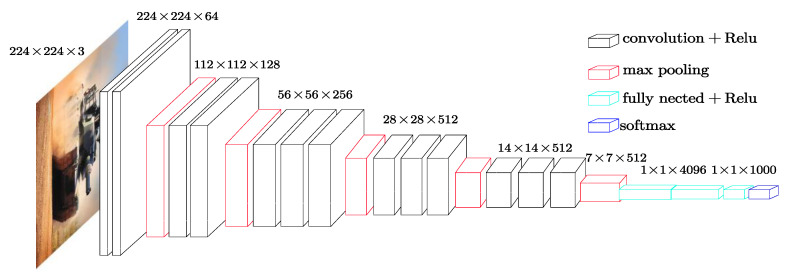
The structure of the Visual Geometry Group (VGG16).

The VGG16 contains 16 weight layers, where 13 are convolution layers and 3 are full connection layers. Figure 8 gives the specific structure of the VGG16. Noting that this network can be divided into different blocks, each block contains several convolutional layers and a pooling layer. Moreover, in the same block, the channel number of the convolutional layer is consistent. With each pooling operation, the width and height of the feature map are reduced by 50% and local information is gradually lost. After five pooling operations, the change process is 224-112-56-28-14-7, while the depth with each convolution doubles: 3-64-128-256-512. The entire process transforms the feature information from the initial 224×224×3 to 7×7×512, and each convolution and pooling operation distributes the local information to different channels.

Compared with other DCNNs, the notable characteristic of the VGG16 is the application of small convolution kernels (1×1, 3×3) and small pooling kernels (2×2). The convolution involves not only calculating works but also receptive fields. The former is related to whether it is convenient to deploy to the mobile terminal, meets the real-time processing, and is easy to train; the latter involves the size of the feature maps, the number of features, and the model complexity. At the same strides, the larger the convolution kernel, the larger the convolution kernel size. As for the receptible field, the authors point out that the receptive field obtained by stacking two 3×3 convolutions is equivalent to a 5×5 convolution, and that stacking three 3×3 convolutions equals a 7×7 convolution. Another advantage is that the fully connected layers are first converted to convolutional layers. Without the full layers, the resulting fully convolutional network is then applied to any size image.

## 5. Experiments

In this section, we present examples that shed light on the stronger ability of our model than that of other comparable methods to accommodate a low SNR. In addition, we also illustrate the excellent performance of the 2D-IVMD technique and show how sharpened images and DCNN can improve performance.

### 5.1. Performance Analysis under Different SNRs

To access the recognition rate and robustness of our methods in the case of a low SNR, Gaussian white noises were added to the echoes. Different noise powers have different effects on recognition rate, and the recognition accuracy degrades with the increasing SNR. In the following experiment, we give the identification results with different SNRs from −10 dB to 2 dB to study the noise suppression ability of the 2D-IVMD method.

The parameters of three typical vehicles and the EM simulation are shown in Table 3. The training and test databases were selected according to the following criteria: (a) the training data cover all of the azimuths from 0 to 180° and the (b) the data set measured at a 30° depression angle is used as library data with 25° being considered as an unknown input.

**Figure 9:**
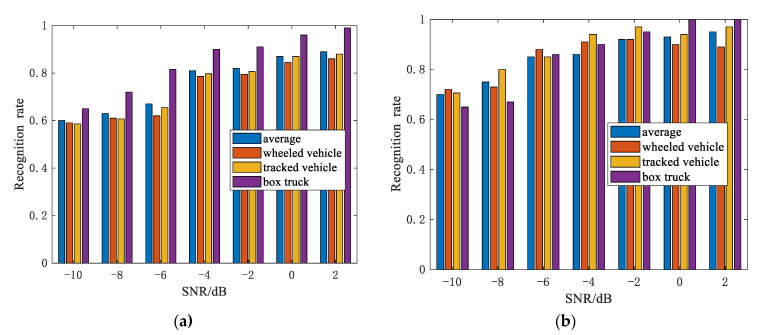
Recognition rate of the three vehicles: (**a**) before the two-dimensional variational mode decomposition (2D-IVMD) procedure and (**b**) after the 2D-IVMD procedure.

Figure 9 shows the recognition accuracy with respect to the different SNR via 100 Monte Carlo trials, where the change interval of the azimuth angles is 10°. As observed, the recognition accuracy significantly improved after the noise was processed by the 2D-IVMD method. The average recognition rate rose as the SNR increased, achieving 0.95 when the SNR was 2 dB. As seen in Figure 9a, the recognition rate of the box truck is higher than that of other vehicles. The reason is the simpler appearance of the box truck and the weaker coiling among the strong scatterings.

### 5.2. The Influence of Decomposition Layers

**Figure 10:**
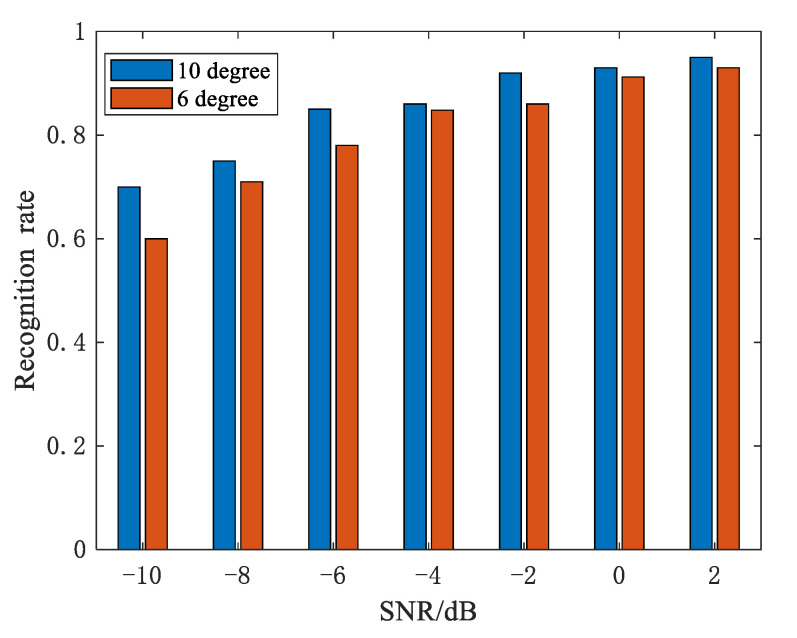
Recognition rate under different change intervals.

**Figure 11:**
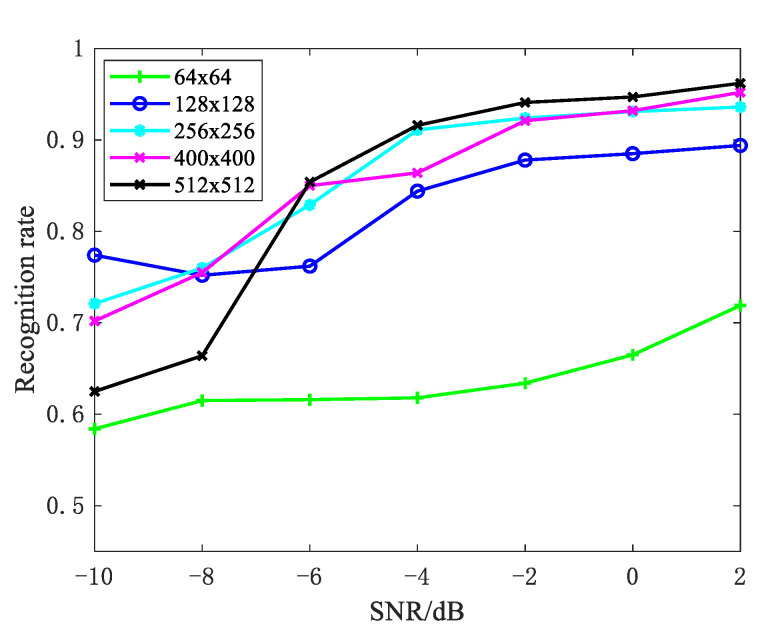
Recognition rate with image sizes.

In the baseline of the experiment in Section 5.1, we confirmed the validity of our method under different SNRs and a 10° change interval for azimuth angles. In this section, we examine the impact of the azimuth angles’ change interval on recognition rate. Looking at the experimental results in Figure 10, when compared to the 6° change interval, the 10° change interval has a better recognition rate. Although wide azimuth angles can better reflect the fluctuating trend of strong scattering points, we must still consider the possibility of obtaining a large continuous observation angle. Therefore, it is critical to maintain a balance between the recognition rate and the actual acquisition. Moreover, image size for CNN is a very important factor that affects the classification performance. Figure 11 gives the recognition rate with different image sizes. We can see that the recognition rate increases as image size increases due to more edge information acquisition, but the conclusion is the opposite on the condition of low SNR. The reason is that a large picture size can obtain edge information while also containing more noise. Additionally, the large image size will increase the computational burden; thus, we need to reach a compromise.

### 5.3. Baselines

In this subsection, we compare our methods with other models related to our works. These models include the principle component analysis (PCA), compressive sensing (CS) [38], and singular value decomposition (SVD) [39] used in the 1D signal processing as well as the 2D empirical mode decomposition (2D-EMD) [40], 2D continuous wavelet transform (2D-CWT) [41], 2D-VMD (the sum of BIMF1 and BIMF2), and SDAE [29] aimed at the 2D signal.

**Figure 12:**
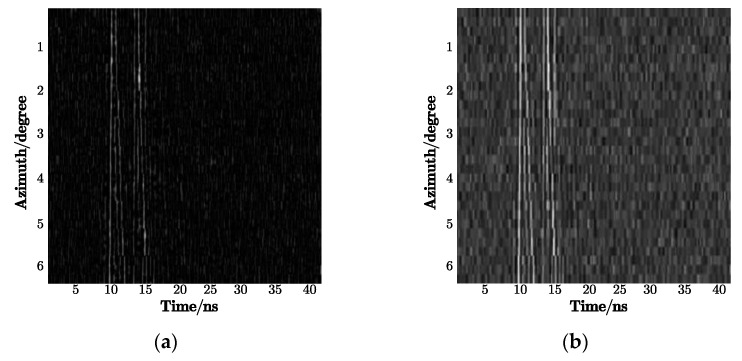
Decomposition results: (**a**) 2D empirical mode decomposition (2D-EMD) and (**b**) 2D-Wavelet.

As shown in Table 4, the 2D-IVMD method has the highest recognition rate among all the techniques, where the change interval of azimuth angles is 10°. Compared with the methods for single-view signals, the algorithms using 2D information had better recognition performance, which is due to the significant differences between 1D echoes for the same target. Moreover, the recognition performance of the 2D-CWT method and the 2D-EMD method was worse than that of the 2D-IVMD method, especially under a low SNR. Figure 12 gives the decomposition results of the 2D-CWT and the 2D-EMD methods. We can clearly see that the noise filtering effect of these methods was relatively poor, and they also attenuated the signal while filtering out the noise. Moreover, the training and test sets were constituted of data at two depression angles, i.e., 30 and 25°, leading to location shifts of scattering centers on the target. The SDAE method cannot be invariant to transformation; thus, the recognition performance is worse than that of 2D-IVMD, but it has better noise robustness on the condition of a low SNR.

## 6. Conclusions

In this paper, we proposed a scheme for complex ground-target recognition based on carrier-free UWB radars for the first time. Different from narrow-band and other traditional UWB radars, the echo signals based on carrier-free UWB radars have extreme bandwidth and no carrier frequency, which increases the difficulty in precise echo waveform acquisition and efficient feature extraction. For these reasons, we established a recognition procedure based on the EM calculation and spectral decomposition. To our best knowledge, we are the first to apply 3ds Max and TDIE to complex ground target modeling, and experiments were subsequently performed to verify the accuracy of the modeling method. Moreover, given the non-linear and non-stationary properties of carrier-free UWB signals, the improved 2D-VMD was proposed as a noise robustness feature extraction method. Rather than only discarding high-frequency components for image denoising, we analyzed the selection of K from the view of the spectra to eliminate noise and preliminarily extract edge features at the same time. Then, transfer learning was introduced to complete the final recognition task. Experimental results demonstrate the effectiveness and generalization of the proposed method under low SNR values. Through this study, we have shown the proposed method can achieve image denoising and sharpening and satisfy certain vision-based applications, such as edge detection, target recognition, and tracking.

## Figures and Tables

**Table 1:** Comparison of the state-of-the-art methods with respect to a number of properties. The + denotes a good performance and the − denotes a bad performance or complete lack thereof.

Model Properties	CWT	EMD	VMD
Decomposition for nonlinear and nonstationary signals	−	+	+
Mode distinction	+	−	+
Mathematical theory	+	−	+
	CNN	RMB	DAE
Feature extraction automatic	+	−	−
Unsupervised learning	−	+	+
Translation invariance	+	−	−
Training efficiency	−	−	+

**Table 2:** Peak signal noise rate (PSNR) with different K.

	2 dB	0 dB	−2 dB	−4 dB	−6 dB	−8 dB	−10 dB
K=2	30.83	28.82	26.86	26.14	25.36	23.99	23.97
K=3	29.90	27.83	25.92	25.02	24.21	22.74	22.73
K=4	28.39	26.63	25.05	24.17	23.38	22.14	21.94
K=5	28.37	26.16	24.49	23.59	22.74	21.52	21.16
K=6	27.94	25.99	24.15	23.47	22.46	21.38	20.84
K=7	27.64	25.66	23.91	23.11	22.17	21.09	20.48
K=8	27.20	25.31	23.88	22.78	21.78	20.75	20.11
K=9	27.05	25.15	23.84	22.63	21.65	20.67	19.91

**Table 3:** Parameters of vehicles and radar in the experiments.

Simulation Parameters	Bandwidth	2.5 GHz	
	Azimuth angles	0–180° with 0.3° steps	
	Depression angles	25°, 30°	
Vehicles	Length (m)	Width (m)	Height (m)
Wheeled vehicles	6.4	2.8	3.1
Tracked vehicles	8.3	3.9	2.4
Box trucks	7.9	3.5	3.2

**Table 4:** Classification performance of the proposed model with several comparable methods.

	2 dB	0 dB	−2 dB	−4 dB	−6 dB	−8 dB	−10 dB
SVD	0.602	0.562	0.544	0.535	0.530	0.525	0.525
CS	0.620	0.591	0.582	0.570	0.517	0.486	0.475
PCA	0.665	0.647	0.632	0.625	0.602	0.600	0.535
Original data + VGG16	0.892	0.883	0.821	0.810	0.788	0.646	0.506
2D-EMD + VGG16	0.890	0.850	0.840	0.835	0.795	0.752	0.624
2D-CWT + VGG16	0.912	0.905	0.832	0.784	0.760	0.722	0.598
SDAE	0.928	0.911	0.895	0.861	0.848	0.806	0.719
2D-VMD + VGG16	0.940	0.924	0.902	0.853	0.800	0.662	0.650
2D-IVMD + VGG16	0.950	0.932	0.921	0.864	0.850	0.795	0.702

## Data Availability

Not applicable.
